# Effects of conventional nursing in the operating room combined with transcutaneous electrical acupoint stimulation on postoperative cognitive dysfunction after total knee arthroplasty in elderly patients

**DOI:** 10.1186/s13018-023-04348-6

**Published:** 2024-02-01

**Authors:** Xinxin Wang, Jia Zhou, Gaojiao Zhang

**Affiliations:** 1 Department of Operation Room, Wenzhou Hospital of Integrated Traditional Chinese and Western Medicine, No. 75 of Jinxiu Roud, Lucheng District, Wenzhou, 325000 Zhejiang People’s Republic of China; 2 Department of Anesthesiology, Wenzhou Hospital of Integrated Traditional Chinese and Western Medicine, No. 75 of Jinxiu Roud, Lucheng District, Wenzhou, 325000 Zhejiang People’s Republic of China

**Keywords:** Conventional nursing in operating room, Inflammatory factors, Postoperative cognitive dysfunction, S100β protein, Transcutaneous electrical acupoint stimulation

## Abstract

**Background:**

To observe the effects of conventional theatre nursing combined with transcutaneous electrical acupoint stimulation (TEAS) on postoperative cognitive dysfunction (POCD) in elderly total knee arthroplasty (TKA) patients.

**Methods:**

Forty elderly TKA patients were randomly divided into a conventional nursing (control) group and a TEAS group. Using conventional nursing, TEAS was used to stimulate the “Zusanli” and “Sanyinjiao” in the healthy leg of patients in the TEAS group. All patients received mini-mental (MMSE) scores 1 day before surgery (T0) and 1, 3, and 7 days after surgery (T1, T3, T7). Plasma levels of interleukin-1 (IL-1β), interleukin-6 (IL-6), tumor necrosis factor (TNF-α), and S100β were measured using venous blood samples.

**Results:**

There were no significant differences in baseline clinical characteristics between the two groups. Compared to T0, the MMSE scores of patients in the control group were significantly reduced at T1 and T3 (*P* < 0.05). Compared to the control group, the MMSE scores of patients in the TEAS group increased significantly at T3 (*P* < 0.05). The incidence of POCD in the TEAS group was 10%, lower than in the control group (40%) (*P* < 0.05). The levels of IL-1β, IL-6, TNF-α and S-100β in patients in the TEAS group were lower than in the control group on days T1, T3 and T7 (*P* < 0.05).

**Conclusion:**

Conventional intraoperative nursing combined with TEAS can reduce the incidence of POCD. The possible mechanism is related to the reduction of inflammatory response and neuronal injury with TEAS.

*Clinical registration number*: ChiCTR2300070281.

## Background

Total knee arthroplasty (TKA) is a highly effective treatment for patients with severe knee dysfunction, with significant improvements in joint function, joint pain, and range of motion [1]. Postoperative cognitive dysfunction (POCD) is a serious complication associated with TKA in elderly patients [2]. POCD has been shown to increase the length of hospital stay and is strongly associated with postoperative quality of life [3]. Therefore, prevention of POCD has become a major challenge for an increasing number of elderly patients. Studies have shown that there is a direct relationship between the incidence of POCD and nerve injury caused by an inflammatory response in the body [4]. However, there is no specific treatment for POCD currently. Acupuncture is a minimally invasive, convenient, and feasible novel therapy. A large number of studies have confirmed that acupuncture can significantly inhibit the inflammatory response of the body [5, 6], and has protective effects on nerve cells [7]. Transcutaneous electrical acupuncture (TEAS) is a non-invasive and simple alternative to acupuncture, combining the advantages of acupuncture and acupuncture with percutaneous electrical nerve stimulation [8]. The application of TEAS clinically can inhibit the stress response of the body to surgery and anesthesia and protect organs [9]. Other studies have shown that appropriate care in the operating room can significantly reduce the incidence of POCD in elderly patients after surgery [10]. Therefore, the present study was conducted to observe conventional care combined with TEAS during surgery and its effects on the incidence of POCD in elderly patients after TKA. Plasma IL-1β, IL-6, TNF-α, and S100β levels were also observed and the possible mechanism was evaluated.

## Methods

### Clinical data

A total of 40 patients who underwent TKA surgery were admitted to the Department of Arthroscopy, Wenzhou Hospital of Integrated Traditional Chinese and Western Medicine from August 2022 to August 2023. The 40 patients were randomly divided into two groups: the conventional care in the operating room (control) group and the TEAS group, with 20 cases in each group. This study was approved by the Ethics Committee of Wenzhou Hospital of Integrated Traditional Chinese and Western Medicine. All patients participating in this study signed a written informed consent with the research institution. The trial was registered at Chinese Clinical Trial Registry on 07/04/2023 with registration number ChiCTR2300070281.

### Inclusion and exclusion criteria

Inclusion criteria were as follows: Patients (male or female) older than 65 years of age; American Society of Anesthesiologists (ASA) class I–II patients; duration of TKA was between 60 and 120 min. Patients who volunteered to participate in this clinical trial and signed an informed consent form.

The exclusion criteria were as follows Patients with severe central nervous system disease such as cerebral haemorrhage and stroke sequelae; patients with mental or emotional illness; patients with a history of severe heart and lung disease; patients who have difficulty communicating with the physician and cannot complete the Cognitive Function Rating Scale due to low educational level; patients with contraindications to combined lumbar epidural anaesthesia and surgery for more than 120 min, lower extremity nerve injury, skin ulceration around acupoints, and no anti-inflammatory medication 1 week before surgery.

### Treatment protocol

Patients had to fast for 8 h and abstain from alcohol for 6 h before surgery. During surgery, venous access was established and ECG monitoring was performed. The patient was placed in lateral decubitus position and combined lumbar and epidural anaesthesia was performed at 2–3 lumbar levels. When CSF outflow was observed during lumbar needle insertion, 3 ml of 0.5% ropivacaine solution was slowly pushed into the subarachnoid space and then the epidural catheter was slowly inserted. During surgery, 0.5% ropivacaine solution was added for epidural anaesthesia according to the patient's surgical situation. The patient's sensory block level remained below the chest throughout the procedure. During surgery, if the patient's blood pressure was less than 20% of the preoperative blood pressure, ephedrine 5 mg was administered sequentially until the blood pressure was corrected to normal. If the heart rate was below 50 beats/min, atropine 0.5 mg was administered until the heart rate was corrected to normal. The number of administrations was recorded. The postoperative intravenous analgesia pump was administered according to the patient's body weight.

Patients in the control group received high quality care in the operating theatre: (1) Pre-operative visits to patients were robust and an appropriate environment was created during surgery to prevent post-operative anxiety for patients. (2) Intra-operative patient care was robust to reduce anxiety and ensure patient safety during surgery. 3. Postoperative patient care and adverse reactions were handled with care.

During surgery, patients in the TEAS group received high quality care in the operating room, similar to patients in the control group. In addition, the “Zusanli” and “Sanyinjiao” points on the lower limb of the healthy side of patients in the TEAS group were attached with electrode stickers[9] and stimulated (dense wave with a frequency of 2 Hz/15 Hz). Stimulation was applied for 30 min before surgery and continued during surgery until the end of the procedure. The intensity was adjusted according to the maximum intensity that the patients could tolerate.

### Observation index

Baseline clinical characteristics including name, sex, age, height, weight, years of education and ASA classification of each group were recorded preoperatively. Blood pressure was measured in all patients on admission, 5 min after anaesthesia, 60 min during surgery and immediately after surgery. Mean arterial pressure (MAP) values and heart rate (HR) were recorded, and operative time, tourniquet time, fluid volume, and blood loss during surgery were noted.

Assessment of cognitive function: The same professionally trained theatre nurse used the Mini-Mental State Examination (MMSE) to assess the patients’ cognitive function at 8:00 am preoperatively and postoperatively on days 1, 3 and 7. POCD was diagnosed when the postoperative MMSE score was 2 points lower than the preoperative baseline score.

Serum levels of IL-1β, IL-6, TNF-α and S100β were determined: Venous blood was collected from patients at 8:00 am preoperatively on day 1 and postoperatively on days 1, 3, and 7. IL-1β, IL-6, TNF-α and S100β levels were determined by enzyme-linked immunosorbent assay (ELISA).

### Statistical analysis

SPSS 25.0 (IBM Corporation, Armonk, NY, USA) was used for statistical analysis, and data were expressed as mean ± standard deviation ($\overline{x}$ + SD). Comparison of male to female constituent ratio and vasoactive drug use was done using Fisher’s exact probability method. Age, BMI, tourniquet use duration, surgery duration, blood loss, and other data were tested using T-test. Repeated measures of ANOVA were used to compare continuous data. Differences with *P* < 0.05 were statistically significant.

## Results

### Comparison of baseline clinical characteristics

A total of 40 patients underwent received TKA surgery was included in this study. The study group consisted of 20 patients with a mean age of (70.2 ± 4.2) years old, 12 males and 8 females. The control group consisted of 20 patients with a mean age of (72.3 ± 5.4) years old, 10 males and 10 females. There were no significant differences between the two groups in gender, age, body mass index (BMI), years of education, ASA classification, operation time, duration of tourniquet use, intraoperative fluid volume, bleeding volume and frequency of ephedrine and atropine use (all *P* > 0.05) (Table 1).

**Table 1:** Comparison of baseline clinical characteristics between the two groups (x̅ + SD).

Clinical characteristics	TEAS group (n = 20)	Control group (n = 20)	*P*
Males/female	12/8	10/10	0.37
BMI (kg/m^2^)	64.36 ± 9.08	64.13 ± 9.21	0.70
Age (years)	70.2 ± 4.2	72.3 ± 5.4	0.31
Years of education	6 ± 2.5	5.7 ± 2.1	0.56
ASA grades			
I	6	5	0.62
II	14	15	
Combined with hypertension	12	13	0.74
Combined with Type 2 diabetes	10	11	0.75
Intraoperative fluid volume (ml)	2250 ± 57.9	2570 ± 80.9	0.21
Bleeding volume (ml)	232 ± 35.2	241 ± 29.1	0.22
Operation time (min)	92.1 ± 10.5	98.5 ± 12.7	0.36
Tourniquet (min)	32 ± 10.9	36 ± 10.1	0.09
Use of ephedrine	15	18	0.46
Use of atropine	5	8	0.36

*TEAS* Transcutaneous acupoint electrical stimulation, *BMI* Body mass index, *ASA* American Society of Anesthesiologists.

### Comparison of MAP and HR in each time period

There was no statistical significance in MAP and HR between the two groups in each time period (all *P* > 0.05) (Table 2).

**Table 2:** Comparison of MAP and HR between the two groups (x̅ + s)

Indicators	Timing	TEAS group (n = 20)	Control group (n = 20)	*P*
MAP	The time of entry	93 ± 11	98 ± 12	0.28
	5 min after anesthesia	85 ± 12	83 ± 15	0.51
	60 min during the operation	95 ± 13	93 ± 10	0.44
	The end of the operation	98 ± 14	97 ± 13	0.74
HR	The time of entry	76 ± 6	78 ± 13	0.54
	5 min after anesthesia	66 ± 9	63 ± 7	0.10
	60 min during the operation	62 ± 11	65 ± 10	0.21
	The end of the operation	52 ± 12	56 ± 9	0.24

*TEAS* Transcutaneous acupoint electrical stimulation, *MAP* Mean arterial pressure, *HR* Heart rate

### Comparison of MMSE scores between the two groups

**Fig. 1:**
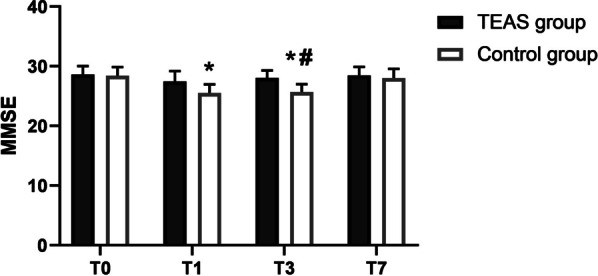
Histogram of MMSE score comparison between the two groups. Abbreviation: Transcutaneous acupoint electrical stimulation (TEAS) group; conventional nursing in operation room (control) group; 1 day before surgery (T0), 1 day after surgery (T1), 3 days after surgery (T3); 7 days after surgery (T7). All data are expressed as the mean ± SD. *P < 0.05, suggesting that comparison with T0 within each group. ^#^*P* < 0.05, suggesting that comparison between the study group and the control group at the same T point

The MMSE score of patients in the control group decreased significantly on the 1st and 3rd day after surgery as compared to the day before surgery (*P < 0.05), while the MMSE score on the 7th day post-surgery exhibited no significant changes compared with the day before surgery. There was no significant change in MMSE score of the TEAS group on the pre-operative day 1 and 1st, 3rd, 7th days after surgery. Patients in the TEAS group had higher MMSE scores than those in the control group 3 days after TKA, and the difference was statistically significant (Fig. 1, ^#^*P* < 0.05).

### Comparing incidences of POCD

POCD occurred in 2 patients in the TEAS group (10%) and 8 patients in the control group (40%). The POCD incidence in the TEAS group was significantly lower than that in the control group (*P* = 0.028).

### Comparison between levels of IL-1β, IL-6, and TNF-α in serum

**Fig. 2:**
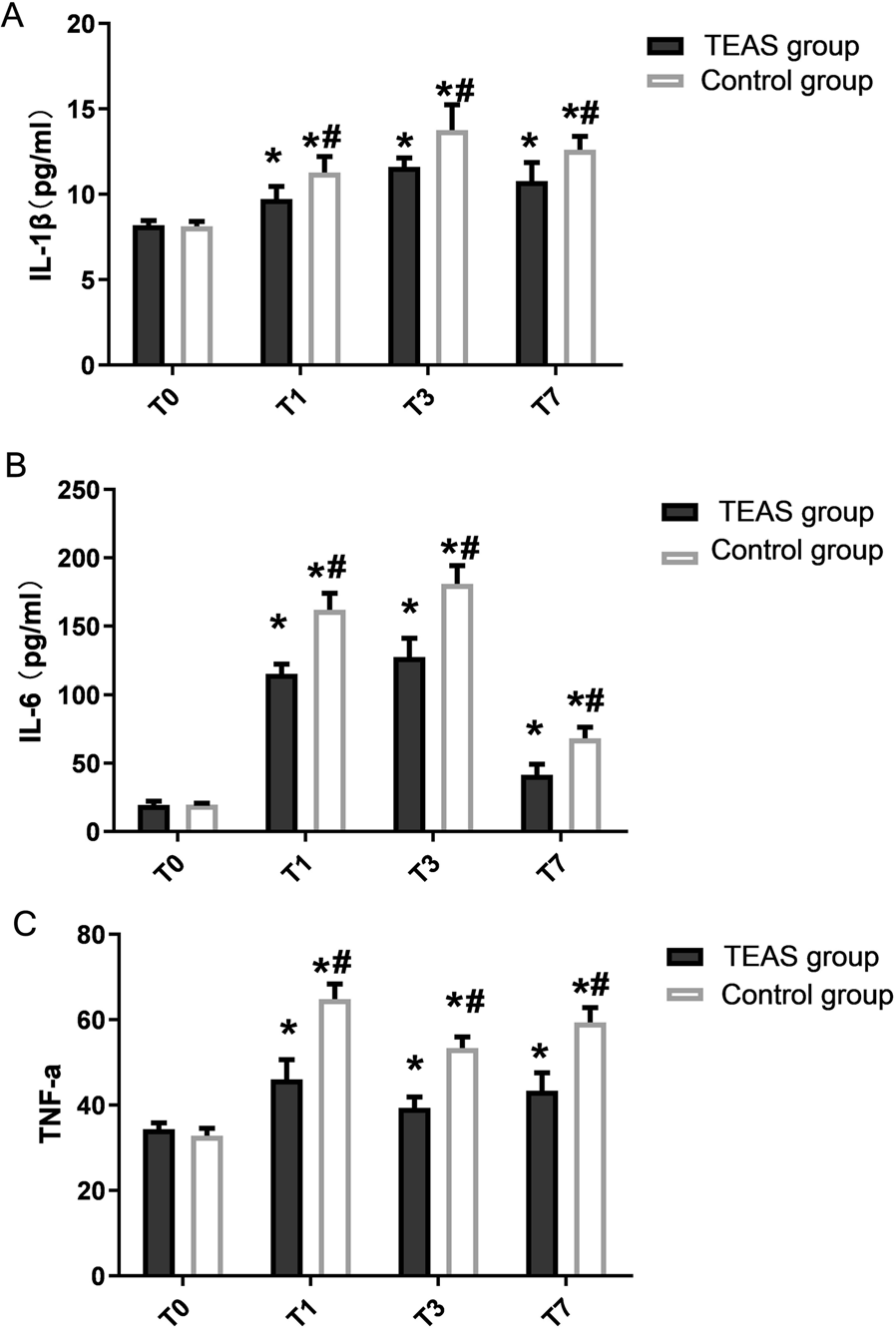
Histogram of the levels of (2A), IL-6(2B) and TNF-α(2C) in serum between the two groups. Abbreviation: Transcutaneous acupoint electrical stimulation (TEAS) group; conventional nursing in operation room (control) group; 1 day before surgery (T0), 1 day after surgery (T1), 3 days after surgery (T3); 7 days after surgery (T7). All data are expressed as the mean ± SD. *P < 0.05, suggesting that comparison with T0 within each group. ^#^*P* < 0.05, suggesting that comparison between the study group and the control group at the same T point

On the pre-operative day 1, there were no significant differences between the two groups in the serum levels of IL-1β, IL-6, and TNF-α. Compared to the pre-operative day 1, the levels of IL-1β, IL-6, and TNF-α in serum in the two groups significantly increased postoperatively on days 1, 3, and 7, and was statistically significant (*P < 0.05). Comparing the two groups, the serum levels of IL-1β, IL-6, and TNF-α in the TEAS group were all lower than those in the control group postoperatively on days 1, 3, and 7, and the difference was statistically significant (Fig. 2A–C, ^#^*P* < 0.05).

### Comparison of S100β levels in serum

**Fig. 3:**
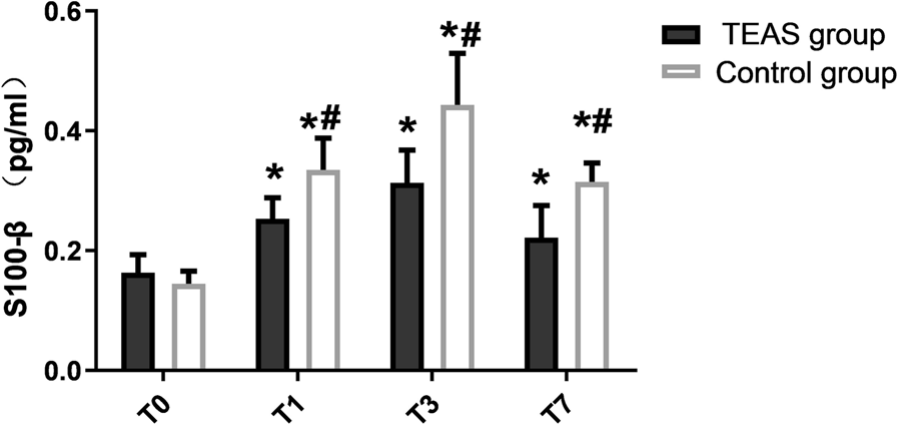
Histogram of the level of S-100β in serum between the two groups. Abbreviation: Transcutaneous acupoint electrical stimulation (TEAS) group; conventional nursing in operation room (control) group; 1 day before surgery (T0), 1 day after surgery (T1), 3 days after surgery (T3); 7 days after surgery (T7). All data are expressed as the mean ± SD. *P < 0.05, suggesting that comparison with T0 within each group. ^#^*P* < 0.05, suggesting that comparison between the study group and the control group at the same T point

Preoperatively on day 1 there was no significant difference in the serum levels of S100β between the two groups. Compared to the preoperative day 1, the level of S100β in serum increased significantly in the two groups post-operatively on days 1, 3, and 7, and the difference was statistically significant (*P < 0.05). Compared to the control group, the serum S100β levels significantly decreased in the TEAS group postoperatively on days 1, 3, and 7, and the difference was statistically significant. (Fig. 3, ^#^*P* < 0.05).

## Discussion

POCD is a common post-surgical impairment that has a severe negative impact on the quality of life of patients, especially the elderly. It manifests as reduced learning ability, memory function, information processing ability and speed, personality change and reduced social activities, accompanied by damage to the central nervous system [11]. Several studies have shown that orthopaedic surgery and elderly patients are two important risk factors for POCD [3]. Therefore, elderly patients undergoing TKA were selected as the experimental subjects in this study.

With the popularity of anaesthesia and surgery, POCD has received increasing attention worldwide. In recent years, nursing has played a very important role in the management of POCD. Nurses should be more aware of the possible impact of various factors on patients, observe them throughout the perioperative period, and evaluate their response to hospitalisation and treatment. This will help to better identify patients who may have POCD and plan preventative interventions to improve their recovery and/or quality of life [10]. Some researchers have used the Neuman System Model to show that the involvement of family members in the care of patients with mental health problems, such as delirium, is effective in increasing patient resilience and has an anti-POCD effect [12]. Thus, the development of medical humanities has led to higher requirements for the safe treatment of POCD. Acupuncture has proven to be an effective treatment for many nervous system diseases [13]. Therefore, in this study, TEAS intervention was used to observe the incidence of POCD in elderly patients undergoing TKA surgery. We observed that the MMSE scores of the control group were significantly lower postoperatively on days 1 and 3 than preoperatively on day 1. While the MMSE score did not decrease after “Zusanli” and “Sanyinjiao” TEAS were performed on the healthy side, 30 min before surgery. At the same time, the MMSE scores in the TEAS group were significantly higher than those in the control group post-operatively on day 3. At the same time, we also statistically determined the incidence of POCD, and found that it was 40% in patients in the control group, which is in line with previous research results [14]. The incidence of POCD in the TEAS group was 10%, significantly lower than that in the control group. The experimental results show that the intervention method of basic nursing combined with TEAS in the operating room effectively prevents the occurrence of POCD after surgery.

Currently, the pathogenesis of POCD is not completely understood. It has been suggested that systemic inflammation and neuroinflammation-induced damage of nerve cells, especially hippocampal neurons play a very important role in the pathophysiological process of POCD [15–17]. These inflammatory reactions may be induced by peripheral surgical trauma or anesthesia, and suppressing them may be the key point in the prevention and treatment of POCD. It has been reported that serum inflammatory cytokines IL-6, IL-1β, and TNF-α are directly related to postoperative POCD in patients [16, 18]. In this trial, we observed that the levels of IL-1β, IL-6, and TNF-α in serum significantly increased in the two groups of patients on the post-operation day 1, day 3, and day 7. However, compared with the levels on the pre-operation day 1, the extent of increase in the TEAS group was significantly lower than that in the control group. These results indicate that TEAS treatment could inhibit the inflammatory response in the body. The change of S100β protein content is an indicator of early brain injury and can be used to judge the severity of POCD in patients [17, 19]. In this study, the serum level of S100β in the two groups significantly increased, but compared to the preoperative level, the increase in the TEAS group was also significantly lower than that in the control group on post-operative days 1, 3, and 7. Like neuromuscular electrical stimulation (NMES), TEAS can increase muscle strength in elderly patients, thereby increasing the stress response to surgery, which may be the mechanism by which TEAS alleviates cognitive dysfunction after TRK [20]. TEAS treatment had a protective effect on nerve cells in elderly patients with TKA and inhibited the inflammatory response in the body, while reducing the release of inflammatory cytokines TNF-α, IL-6, and IL-1β, thus reducing nerve cell damage (reduced S100β content).

## Conclusions

In conclusion, TEAS could reduce the incidence of POCD and the plasma levels of IL-1β, IL-6, TNF-α, and S100β in elderly patients with TKA, and the possible mechanism is related to a reduction in the inflammatory response and nerve cell injury in the body through TEAS.

## Data Availability

The data that support the findings of this study are available from the corresponding author, upon reasonable request.

## Abbreviations

COPD: Postoperative cognitive dysfunction
TEAS: Transcutanclus electrical acupoint stimulation
TKA: Total knee replacement
ASA: American Society of Anesthesiologists
ECG: Electrocardiograph
MMSE: Mini-Mental State Examination
ELISA: Enzyme-linked immunosorbent assay
BMI: Body mass index
HR: Heart rate
